# Study on Life History Strategies of Anurans in Tropical Rainforest: Lifespan and Age at Sexual Maturity

**DOI:** 10.1002/ece3.73924

**Published:** 2026-06-30

**Authors:** Rui Guo, Li Zhu, Longhui Zhao, Xiaofei Zhai, Tongliang Wang, Jichao Wang

**Affiliations:** ^1^ Ministry of Education Key Laboratory for Ecology of Tropical Islands, Key Laboratory of Tropical Animal and Plant Ecology of Hainan Province, College of Life Sciences Hainan Normal University Haikou China; ^2^ Diaoluo Mountain Diversity of Vertebrate Hainan Observation and Research Station Lingshui Hainan China

## Abstract

This study investigates the life history strategies of tropical anurans on Hainan Island, focusing on lifespan and age at sexual maturity. Seven common species from Diaoluoshan National Forest Park were examined, including *Duttaphrynus melanostictus* , *Amolops torrentis* , *Odorrana graminea* , *Odorrana hainanensis* , *Polypedates megacephalus* , *Kurixalus odontotarsus* , and *Fejervarya multistriata* . Significant interspecific variation was observed in lifespan and age at sexual maturity. Most species, including *D. melanostictus* , *A. torrentis* , *O. graminea* , *P. megacephalus* , *K. odontotarsus* , and *F. multistriata* , reach sexual maturity at 3 years old, with lifespans ranging from 5 to 9 years. Species such as *O. hainanensis* matured later, at 4 years old, and exhibited longer lifespans. The study also revealed distinct life history strategies, with most species allocating approximately 50% of their lifespan to growth and 50% to reproduction. However, species like *A. torrentis* and *K. odontotarsus* devote 60% of their lifespan to growth, while *O. hainanensis* allocates 56% of its lifespan to reproduction. These strategies reflect adaptations to the stable climatic conditions of tropical rainforests, where resources are abundant and predation pressure is lower, allowing for slow‐life history strategies. These findings provide valuable insights into the adaptive strategies of tropical amphibians and underscore the importance of stable tropical environments in shaping their life history evolution. The results also contribute to amphibian conservation strategies and ecological management in tropical rainforest ecosystems.

## Graphical abstract

This study presents the first comprehensive analysis of lifespan and age at sexual maturity in seven common anuran species from Hainan Island's tropical rainforest, filling a critical gap in tropical amphibian ecology. The results reveal significant interspecific variation in lifespan (5 ~ 9 years) and maturity age (3 ~ 5 years), indicating a “slow‐life” strategy with delayed maturity and extended reproductive investment among these tropical anurans.

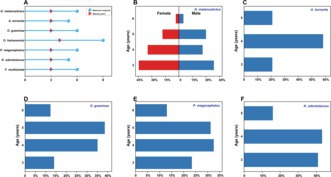

## 1 Introduction

Life history strategy is a foundational framework in evolutionary ecology, which elucidates how organisms optimize energy allocation to balance growth, survival, and reproduction under specific environmental pressures. Lifespan and age at sexual maturity are pivotal determinants within this framework, as their trade‐offs reflect species' adaptive strategies to environmental constraints. For amphibians, these trade‐offs illustrate how individuals allocate resources to maximize fitness across dynamic ecological conditions (Stearns 1998; Hayes 2009).

Amphibians, characterized by their high sensitivity and adaptability to environmental changes, have become pivotal model organisms for studying life history strategy. Investigating amphibian life history strategies holds significant practical relevance for biodiversity conservation. Global threats such as climate change, habitat loss, environmental pollution, and diseases like chytridiomycosis are profoundly affecting life history strategies of amphibian populations (Woodhams et al. 2008; Scheele et al. 2017; Becker et al. 2018; Turner et al. 2022). Understanding amphibians' lifespan and maturation strategies provides essential data for predicting population responses to environmental shifts and developing effective conservation measures (Song et al. 2022; Gould et al. 2022; Huang et al. 2023). Amphibians exhibit remarkable variability and heterogeneity in life history traits; for instance, their lifespan can vary widely, from a few years in smaller species (e.g., *Rana temporaria* ) to several decades in large‐bodied species (e.g., *Andrias japonicus* ) (Hjernquist et al. 2012; Köhler et al. 2023). Delayed maturity and longer lifespans often prevail in stable environments, enabling sustained reproductive output over extended periods, whereas rapid maturation and shorter lifespans dominate in unpredictable or high‐risk environments (Guo et al. 2018; Zhang et al. 2018).

The age at sexual maturity in amphibians is influenced by both intrinsic factors (e.g., genetics, body size) and extrinsic factors (e.g., resource availability, temperature). Species inhabiting resource‐rich or stable environments can delay maturation to allocate more time for growth, enhancing long‐term fitness (Jonsson et al. 2013; Jonsson and Jonsson 2014). Conversely, rapid maturation often occurs in environments with high mortality risks, where earlier reproduction ensures population persistence (Verberk et al. 2008; Guo et al. 2018; Zhang et al. 2018). Such variability highlights the ecological and evolutionary importance of studying amphibian life history strategies to reveal adaptive mechanisms under different environmental conditions.

China has a rich diversity of amphibians, with a total of 735 recognized species, 3 Gymnophiona, 102 Caudata, and 630 Anura species (AmphibiaChina 2025). These species are mainly distributed in provinces such as Yunnan, Sichuan, Guizhou, Guangxi, and Hainan, regions characterized by subtropical monsoon and tropical monsoon climates. Current research on amphibian life history traits in China has predominantly focused on subtropical monsoon climate zones, including Yunnan, Sichuan, Guizhou, and Guangxi, with an emphasis on the effects of altitude on life‐history traits (Liao and Lu 2010; Jiang et al. 2022; Xu et al. 2024). Nevertheless, despite the notable amphibian species richness in tropical regions such as Hainan, which represent a substantial portion of China's amphibian diversity, life history research pertaining to amphibians in these zones remains largely unexplored. As China's only tropical island, Hainan hosts extensive tropical rainforests and wetlands that provide unique habitats for diverse anuran species. These ecosystems constitute a natural laboratory for investigating life history strategies under stable, resource‐abundant conditions, which are distinctly different from those in subtropic or high‐altitude environments.

Based on life‐history theory and prior evidence, we hypothesize that compared to temperate anurans which typically reach sexual maturity at 1–2 years (Liao et al. 2016; Jiang et al. 2022), have short lifespans of 2–5 years (Chen et al. 2011; Lou et al. 2012), and allocate only 25%–35% of their lifespan to growth (Lu et al. 2006; Ma et al. 2009) due to seasonal resource limitation, high environmental variability, and the need to prioritize early reproduction in unpredictable environments. Tropical anurans in Hainan's stable rainforests, which are characterized by abundant resources, low predation pressure, and reduced environmental stress, perhaps exhibit delayed sexual maturity and longer lifespans.

This study aims to address the current research gap by investigating the trade‐offs between lifespan and age at sexual maturity in 7 common anuran species on Hainan Island. Specifically, the study explores how these tropical amphibians balance growth and reproductive investments to adapt to resource dynamics and ecological pressures. Through comparative analysis, this research provides critical insights into the ecological strategies of tropical anurans, contributing to the broader understanding of life history evolution while offering scientific data to support amphibian conservation and tropical ecosystem management.

## 2 Materials and Methods

### 2.1 Study Site

Hainan Island, situated in southern China, is the country's only tropical island, characterized by a humid, rainy climate and a unique geographic environment. The island's tropical rainforests and wetland habitats provide ideal conditions for a diverse array of amphibians. This study focused on Diaoluoshan National Forest Park, a representative tropical rainforest ecosystem, to investigate the life history strategies of anurans by examining their longevity and age at sexual maturity. Diaoluoshan National Forest Park (E: 109°41′38″ ~ 110°4′46″, N: 18°38′42″ ~ 18°50′22″) is located at the junction of Lingshui, Qiongzhong, Baoting, and Wanning counties (cities) in the southeastern part of Hainan Island. It falls within the East Asian maritime tropical monsoon climate zone, characterized by abundant annual rainfall of 1870 to 2760 m. The wet and dry seasons are distinctly divided, with over 80% of the rainfall occurring during the rainy season from May to October each year, while the dry season lasts from November to April of the following year. The average annual temperature is 20.8°C. Based on a synthesis of the above information, Diaoluoshan National Forest Park is characterized by abundant resources and a stable climate.

This study was conducted at the research site located within the Diaoluo mountain diversity of vertebrate Hainan observation and research station (E: 109°51′42″ ~ 109°54′20″, N: 18°41′41″ ~ 18°44′22″, elevation 600–920 m) in the Diaoluo Mountain National Forest Park.

### 2.2 Study Subjects

In this study, the lifespan and age at maturity of 7 amphibian anurans in Daoluoshan National Forest Park were investigated, namely, *Duttaphrynus melanostictus* (♀: *n* = 122, ♂: *n* = 80), *Amolops torrentis* (♀: *n* = 4, ♂: *n* = 48), *Odorrana graminea* (♀: *n* = 4, ♂: *n* = 57), *Odorrana hainanensis* (♀: *n* = 25, ♂: *n* = 11), *Polypedates megacephalus* (♀: *n* = 7, ♂: *n* = 77), *Kurixalus odontotarsus* (♀: *n* = 21, ♂: *n* = 45), and *Fejervarya multistriata* (♀: *n* = 3, ♂: *n* = 18). These 7 species are commonly found in Diaoluo mountain diversity of vertebrate Hainan observation and research station, including the widely distributed species in southern China— *D. melanostictus* , *P. megacephalus* , and *F. multistriata* , as well as the endemic Hainan species such as *A. torrentis* and *O. hainanensis* .

*D. melanostictus* is a common species in the family Bufonidae, widely distributed in the southern regions of China. *A. torrentis* is a small frog species in the family Ranidae and the genus *Amolops*, primarily inhabiting mountain streams and moist forest environments. *O. graminea* is a species in the genus *Odorrana* of the family Ranidae, typically inhabiting moist environments near water and widely distributed in the southern regions of China. *O. hainanensis* belongs to the family Ranidae and the genus *Odorrana*, which often inhabits the rock walls beside waterfalls or the grass by streams, and is an endemic species of Hainan Island. *P. megacephalus* belongs to the family Rhacophoridae and the genus *Polypedates*, which is a typical arboreal frog species, widely distributed in southern China. *K. odontotarsus* belongs to the family Rhacophoridae and the genus *Kurixalus*. This frog inhabits shrubland areas and is primarily distributed in southern China. *F. multistriata* , also known as the rice field frog, belongs to the family Dicroglossidae and the genus *Fejervarya*. This frog primarily inhabits still waters or nearby grasslands and is widely distributed in southern China.

### 2.3 Sample Collection

During reproductive season (from April to August 2019), breeding individuals were randomly captured during field surveys. Sex was determined based on external features, including nuptial pads and vocal sacs. Strict maturity verification was conducted for each captured individual: for females, egg‐bearing status and pairing behavior were examined; for males, secondary sexual characteristics (nuptial pads and vocal sacs) were checked, combined with the observation of courtship calls. These multi‐faceted confirmations ensured that all sampled individuals were sexually mature. Then, body length (snout vent length) was measured and the terminal phalanx of the longest right third toe was clipped and preserved in 4% paraformaldehyde for skeletochronological analysis. The amputated area was disinfected with 0.5% iodine solution to prevent inflammation, and individuals were subsequently released at the site of capture.

### 2.4 Age Determination

The ages of all the captured amphibians were determined using standard skeletochronological methods, which have been used in a variety of vertebrates, including fish, amphibians, reptiles, and other marine vertebrates (Halliday and Verrell 1988; Meunier 2012; Sinsch 2015). Muscle and skin of the toes were removed, and the bone was washed in running tap water for 12 h, decalcified in 5% nitric acid for 12–24 h according to size, washed in running tap water for 12 h, stained with Ehrlich's hematoxylin for 2 h, and then washed in running tap water for 20 min. Cross‐sections (6–8 μm) of the diaphyseal region of the bone were obtained using paraffin sections, and the number of the lines of arrested growth (LAGs) in the sections was counted by using a light microscope. For method validation in the present study: (1) Given the strong seasonal temperature fluctuations in the study area, LAGs were confirmed to form annually, as seasonal environmental changes drive periodic bone growth arrest (Xu et al. 2024; Liao et al. 2016); (2) False lines (fainter and incomplete) and double lines were distinguished from true LAGs, with double lines counted as one annual growth cycle to avoid age overestimation (Jiang et al. 2022); (3) Well‐defined lines were observed in all bone sections, indicating that endosteal resorption did not interfere with age determination (Xu et al. 2024).

### 2.5 Determination of Age at Sexual Maturity and Lifespan

In this study, the samples were all obtained from individuals actively engaged in reproduction, and the amphibian species studied exhibits continuous reproduction throughout life. Therefore, the age at sexual maturity was determined as the minimum age of individuals involved in breeding, while the oldest breeding individual represented the species' lifespan. Because lifespans vary among species, this study employed relative age for comparison in order to assess the allocation of lifespan to growth, survival, and reproduction across different species. Relative age was calculated as follows: relative age = age of individual/maximum lifespan of species. Relative age values range from 0 to 1, normalizing age comparisons across species with varying lifespans (Horvath et al. 2022; Riddle et al. 2024).

### 2.6 Life History Strategy Analysis

To assess trade‐offs between growth and reproduction, the study analyzed the proportion of lifespan allocated to growth (prematurity) and reproduction (postmaturity). Relative age at sexual maturity was used as an indicator to explore the balance between growth investments and reproductive strategies, providing insights into life history adaptations under tropical conditions.

### 2.7 Data Analysis and Visualization

Statistical analyses were conducted using SPSS 20.0 (IBM Corporation). Core parameters, including lifespan, age at maturity, and relative age, were compared across species. This study only conducted statistical analyses on species with a sample size of 25 or more. A one‐way ANOVA test was used to analyze differences in age and snout vent length among different species. Multiple comparison results were corrected using the Bonferroni method, with *p* < 0.05 considered as the significance level for differences. Age structure diagrams were drawn for species with a sample size of 45 or more. Data visualization, including charts and graphs, was created using Origin 24 (Electronic Arts Inc).

## 3 Results

### 3.1 Interspecific Variation in Lifespan and Age at Sexual Maturity of Anurans

**FIGURE 1:**
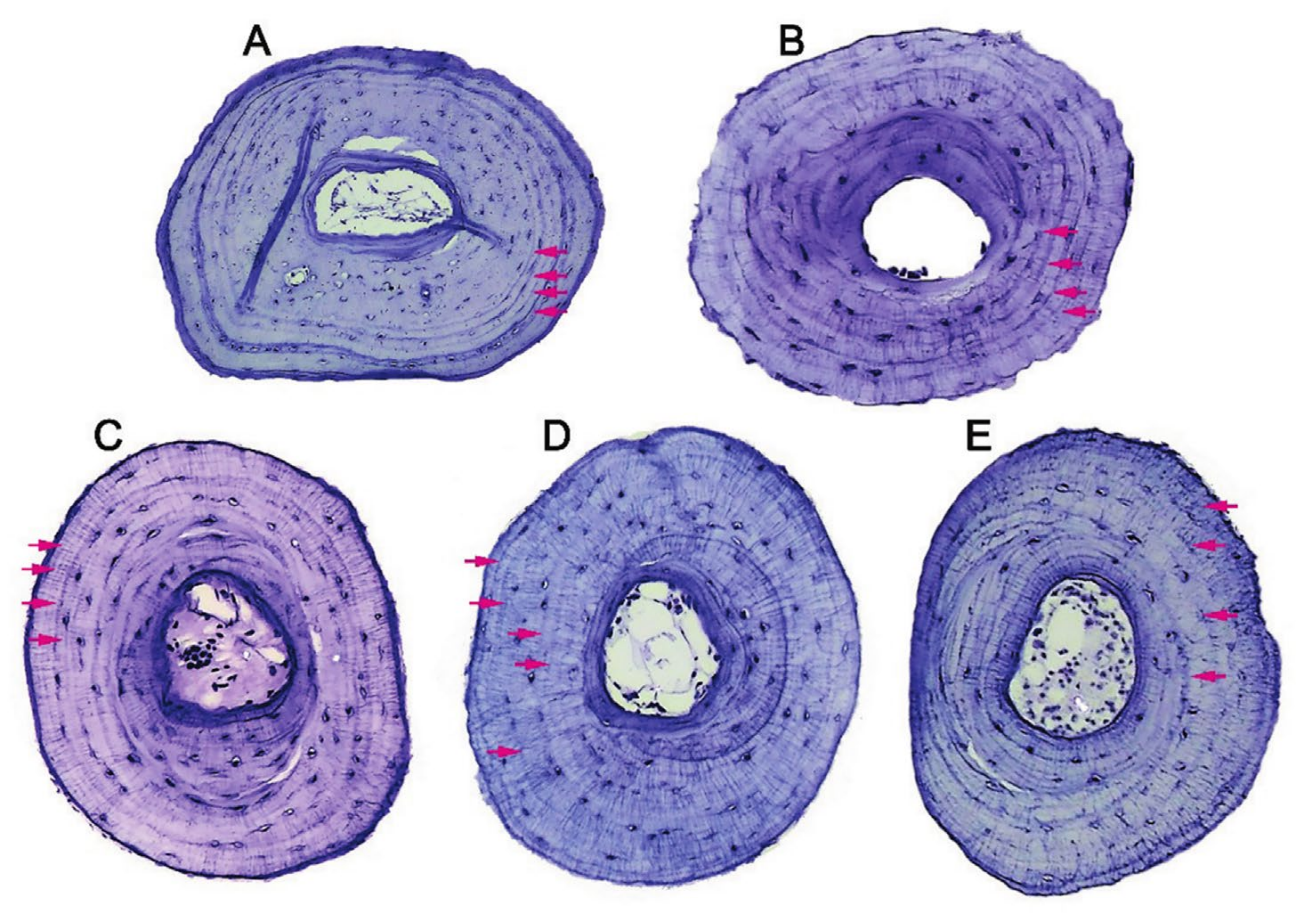
Skeletochronological age of *D. melanostictus* (A), *A. torrentis* (B), *O. graminea* (C), *P. megacephalus* (D), and *K. odontotarsus* (E). The red arrow indicates the lines of arrested growth (LAGs).

**FIGURE 2:**
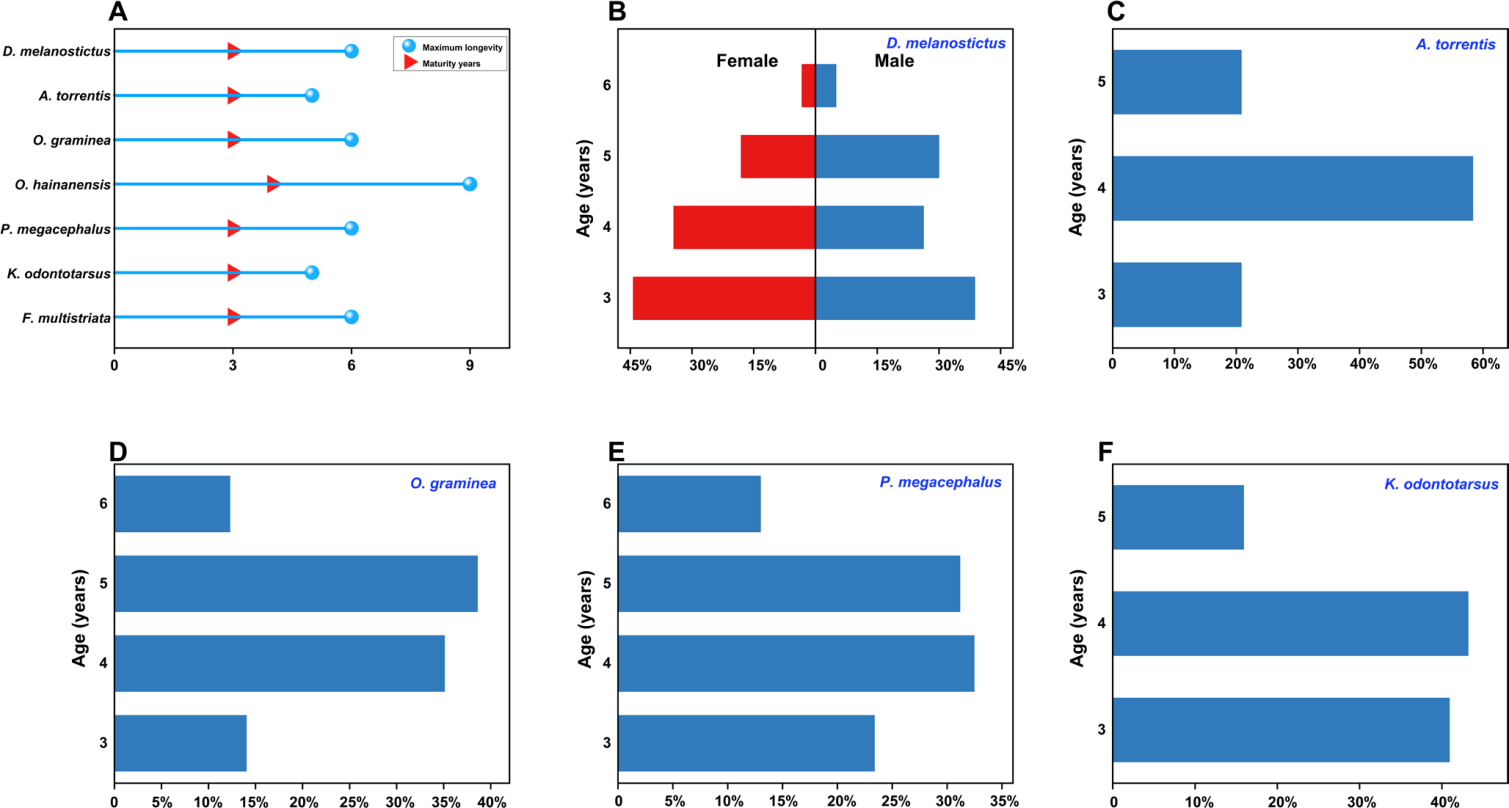
Interspecific variation in age at sexual maturity (the minimum age of breeding individuals), lifespan, and breeding age structure. (A) Age at sexual maturity and maximum lifespan of each species; (B) breeding age structure of *D. melanostictus* ; (C) breeding age structure of *A. torrentis* ; (D) breeding age structure of *O. graminea* ; (E) breeding age structure of *P. megacephalus* ; (F) breeding age structure of *K. odontotarsus* .

This study determined the ages of breeding populations for 7 anuran species using skeletochronological methods (Figure 1). The results found significant interspecific variation in the age at sexual maturity and lifespan of anurans in Diaoluoshan National Forest Park. Most species, including *D. melanostictus* , *A. torrentis* , *O. graminea* , *P. megacephalus* , *K. odontotarsus* , and *F. multistriata* , reached sexual maturity at 3 years old (Figure 2A). Some species reached sexual maturity later, such as *O. hainanensis* , which reached sexual maturity at 4 years (Figure 2A). And then, the longest lifespan of amphibians in Diaoluoshan National Forest Park could reach 9 years old, such as *O. hainanensis* , while the shortest maximum lifespan was 5 years old, as seen in *A. torrentis* and *K. odontotarsus* (Figure 2A). Most amphibians had a maximum lifespan of 6 years old, such as *D. melanostictus* , *O. graminea* , *P. megacephalus* , and *F. multistriata* (Figure 2A).

The study plotted the age structure diagram for breeding populations with a sample size greater than or equal to 45. According to the breeding age structure, the main breeding stage of *D. melanostictus* was between 3 and 5 years old, with few individuals participating in breeding at 6 years old (Figure 2B). The main breeding stage of *A. torrentis* was also between 3 and 5 years old, with most breeding individuals at 4 years old (Figure 2C). For *O. graminea* , the main breeding stage was between 3 and 6 years old, with a larger number of breeding individuals at 4 and 5 years old (Figure 2D). The main breeding age for *P. megacephalus* was between 3 and 6 years old (Figure 2E). The *K. odontotarsus* mainly bred between 3 and 5 years of age (Figure 2F).

Integrating the above findings, this study revealed that anurans in the Diaoluoshan National Forest Park exhibited significant interspecific variation in both the age at sexual maturity and lifespan. Most species reached sexual maturity at 3 years old, while some mature later, with the maximum lifespan recorded being 9 years old. The primary reproductive period for these species generally spanned from 3 to 6 years of age, with the peak breeding age varying among species.

### 3.2 Interspecific and Sexual Variations in Age and Body Size Among Breeding Anurans

**FIGURE 3:**
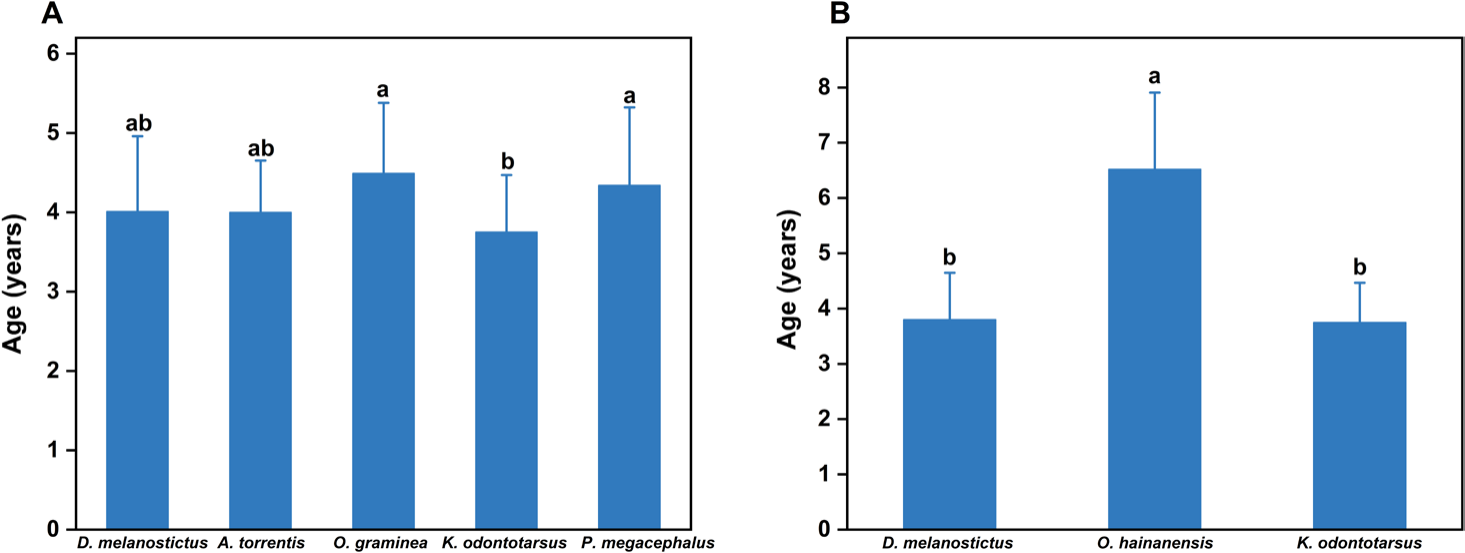
Average age variation among breeding anurans. (A) Male, and (B) Female.

To ensure the accuracy of the results, this study only compared species with a sample size greater than or equal to 25. By analyzing the differences in the average age during the breeding period among the species, the results revealed that, in males, the average age from highest to lowest was as follows: *O. graminea* , *P. megacephalus* , *D. melanostictus* , *A. torrentis* , and *K. odontotarsus* (Figure 3A). Among these, the male *O. graminea* had the highest average age (4.5 years old), followed by *P. megacephalus* (4.3 years old), both of which were significantly older than the *K. odontotarsus* (*p* < 0.05) (Figure 3A). In females, *O. hainanensis* had the highest average age (6.5 years old), followed by *D. melanostictus* (3.8 years old) and *K. odontotarsus* (3.8 years old), with *O. hainanensis* showing a significantly higher average age than both *D. melanostictus* and *K. odontotarsus* (*p* < 0.05) (Figure 3B).

Meanwhile, this study provided a comprehensive assessment of body length across multiple amphibian species and age classes. In *D. melanostictus* , pronounced sexual dimorphism was observable across age classes 3 to 6, with females displaying consistently larger mean snout vent length and greater variability relative to males (Table 1). For example, 5‐year‐old females averaged 78.28 ± 11.62 mm, significantly greater than the male mean of 68.05 ± 5.56 mm (*p* < 0.05) (Table 1).

**TABLE 1:** Body length data of each species during the breeding period.

Species	Sex	Snout vent length (mm) (mean ± SD, minimum~maximum)					
		3	4	5	6	7	8
***D. melanostictus***	Female	69.41 ± 10.83 47.82 ~ 97.22	73.41 ± 12.09 52.77 ~ 100.70	78.28 ± 11.62^a^ 63.34 ~ 98.83	79.20 ± 8.67 71.71 ~ 91.46		
	Male	64.42 ± 5.64**a** 46.13 ~ 74.92	65.72 ± 5.36**a** 56.82 ~ 76.87	68.05 ± 5.56**a** 56.94 ~ 79.11	69.36 ± 3.84**a** 64.18 ~ 73.17		
***A. torrentis***	Male	29.77 ± 1.69**b** 28.26 ~ 33.98	30.17 ± 1.61**b** 26.94 ~ 33.80	30.51 ± 1.59**b** 28.25 ~ 33.54			
***O. graminea***	Male	46.40 ± 2.38**c** 42.89 ~ 49.89	47.25 ± 2.02**c** 44.01 ~ 50.75	47.63 ± 2.14**c** 44.39 ~ 52.56	48.75 ± 2.17**b** 44.28 ~ 50.63		
***O. hainanensis***	Female		106.18 ± 15.03^b^ 95.55 ~ 116.81	111.04 ± 9.20^b^ 102.25 ~ 120.61	113.25 ± 6.00^b^ 107.69 ~ 121.07	114.68 ± 1.35 113.31 ~ 116.01	115.74 ± 4.38 110.86 ~ 123.47
***P. megacephalus***	Male	50.92 ± 2.86**c** 46.70 ~ 55.32	49.67 ± 3.29**c** 44.75 ~ 56.72	51.38 ± 2.55**c** 46.93 ~ 56.23	50.13 ± 3.12**b** 45.06 ~ 55.18		
***K. odontotarsus***	Male	31.50 ± 1.75**b** 29.61 ~ 33.07	31.53 ± 1.20**b** 30.68 ~ 32.37	32.81 ± 0.99**bc** 32.11 ~ 33.51			

^a^ Represents significant differences between males and females of *D. melanostictus* .

^b^ Represents significant differences between females of *D. melanostictus* and *O. hainanensis* , and letters represent significant differences between males of different species.

Interspecific comparisons among males of the same age cohort revealed distinct size disparities. At 3 years old, the snout vent lengths of *D. melanostictus* males (64.42 ± 5.64 mm) were significantly larger than those of males *A. torrentis* (29.77 ± 1.69 mm), *O. graminea* (46.40 ± 2.38 mm), *P. megacephalus* (50.92 ± 2.86 mm), and *K. odontotarsus* (31.50 ± 1.75 mm) (*p* < 0.05) (Table 1). This pattern of interspecific variation among males persisted through 4 and 5 years old, with *D. melanostictus* generally remaining the largest, followed by *P. megacephalus* and *O. graminea* , while *A. torrentis* and *K. odontotarsus* were consistently the smallest (Table 1). Among females, 4‐year‐old female *O. hainanensis* (106.18 ± 15.03 mm) was substantially larger than female *D. melanostictus* (73.41 ± 12.09 mm) (*p* < 0.05) (Table 1).

Regarding age‐related growth within each species, most exhibited a general trend of increasing mean snout vent length with age, though the magnitude varies. For example, male *O. graminea* showed a steady increase from 46.40 mm (3 years old) to 48.75 mm (6 years old). In contrast, male *P. megacephalus* showed less pronounced age‐related change, with means fluctuating around 50–51 mm from 3 to 6 years old. The smallest species, *A. torrentis* and *K. odontotarsus* , showed very modest increases in mean snout vent length across their recorded ages (3–5 years old).

In summary, the body length data revealed complex interactions among species, sex, and age. *D. melanostictus* exhibited marked sexual dimorphism, while *O. hainanensis* females were notably large. Significant differences existed among males of different species at the same age, with a consistent ranking in size. Additionally, most species displayed a trend of increasing snout vent length with advancing age.

### 3.3 Life History Strategies of Anurans in Tropical Rainforests

**FIGURE 4:**
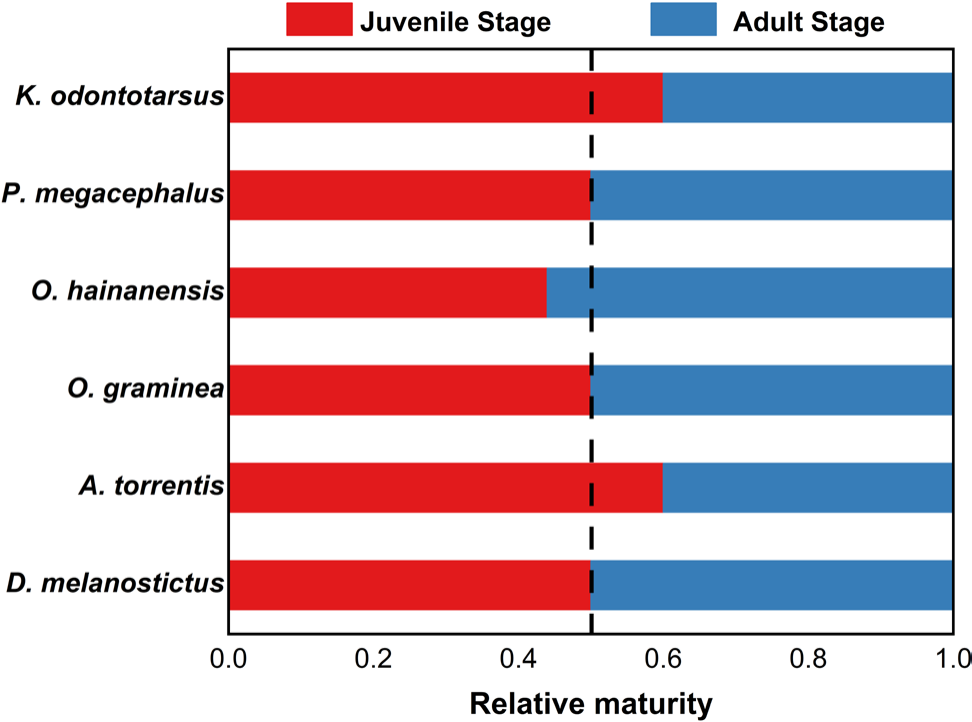
Life history strategy of amphibians in Diaoluoshan National Forest Park.

Analysis of growth and reproductive investments across the lifespan of anurans indicated distinct life history strategies among species in Diaoluoshan National Forest Park, the research results showed that most anurans in Diaoluoshan National Forest Park spent half of their life on growth (prematurity) and the other half on reproduction, such as *D. melanostictus* , *O. graminea* , and *P. megacephalus* (Figure 4). In addition, some amphibian species spent more time on growth, such as *A. torrentis* and *K. odontotarsus* , devoting 60% of their life to growth and 40% to reproduction (Figure 4). Conversely, some species spent more time on reproduction, such as *O. hainanensis* , which spent 44% of its life on growth and 56% on reproduction (Figure 4). In conclusion, the results of this study indicated that anurans in tropical rainforests allocated approximately half of their lifespan to growth and the other half to reproduction.

## 4 Discussion

Despite the remarkable diversity of anurans in China, with 585 documented species, there remains a significant knowledge gap concerning their life history traits. Current data are available for only 24 species across 124 populations, primarily from subtropical and high‐altitude regions (Zhong et al. 2018; Peng et al. 2022). This research addresses this void by focusing on Hainan Island, China's only tropical island, renowned for its unique ecological conditions, including extensive tropical rainforests and wetlands. These habitats provide ideal conditions for diverse anuran species yet remain critically understudied, particularly in terms of lifespan and sexual maturity. This study investigated the life history characteristics of 7 common anuran species ( *D. melanostictus* , *A. torrentis* , *O. graminea* , *O. hainanensis* , *P. megacephalus* , *K. odontotarsus* , and *F. multistriata* ) in the Diaoluoshan National Forest Park, marking the first exploration of the life history traits of anurans on tropical islands.

### 4.1 Species‐Specific Differences in Lifespan and Age at Sexual Maturity

The present study results demonstrated significant interspecific variations in lifespan and age at sexual maturity among the anurans of Diaoluoshan National Forest Park. Due to the limited sample sizes of certain species, such as *O. hainanensis* and *F. multistriata* , the findings for these two species are considered preliminary data for subsequent studies. Therefore, no relevant statistical comparative analyses were conducted in the main text. The results found that lifespan ranged from 5 to 9 years, with *O. hainanensis* exhibiting the longest recorded lifespan (9 years) and *A. torrentis* and *K. odontotarsus* having the shortest (5 years). Similar trends had been observed in other amphibian populations, where lifespans can vary substantially both between species and across geographical populations (Liao and Lu 2010; Liao et al. 2016; Jiang et al. 2022). For instance, temperate species such as *Scutiger boulengeri* (15 years) and *Feirana quadranus* (12 years) exhibited greater longevity, whereas smaller species like *Rana chensinensis* and *Pelophylax pleuraden* lived only 2 ~ 3 years (Chen et al. 2011; Lou et al. 2012). The differences between species in our study underscore the role of ecological and evolutionary pressures in shaping life history traits.

The results of this study indicated that most anuran species in the Diaoluoshan National Forest Park reach sexual maturity at 3 years of age, such as *D. melanostictus* , *A. torrentis* , *O. graminea* , *P. megacephalus* , *K. odontotarsus* , and *F. multistriata* . In addition, *O. hainanensis* reached sexual maturity at 4 years old. According to existing reports, the age at sexual maturity for most anuran amphibians distributed in China was 1 ~ 2 years (Liao et al. 2016; Jiang et al. 2022). For example, male *P. megacephalus* from the Fanjing, Changning, Shangzhong, Jinyun, and Leigong mountains in western China reached sexual maturity at 2 years old, and the lifespan was 5 years old (Jin et al. 2017). Compared with anurans in other regions of China, anurans in tropical rainforests delayed sexual maturity and highlight a distinct “slow‐life” strategy (Ma et al. 2009; Yu et al. 2018; Jiang et al. 2022). These species delayed reproduction and invest more time in growth and survival, which is typical of stable tropical environments with lower predation pressures and abundant resources (Jonsson et al. 2013; Jonsson and Jonsson 2014).

### 4.2 Life History Strategies and Growth‐Reproduction Trade‐Offs

The trade‐off between growth and reproduction is a cornerstone of life history theory, reflecting evolutionary adaptations to environmental pressures (Stearns 1998; Wells 2019). Existing studies have demonstrated that most temperate anurans adopt a fast life‐history strategy, whereby approximately 25%–35% of their lifespan is allocated to growth and the remainder to reproduction (Supplementary Table S1). For instance, *R. chensinensis* reaches sexual maturity at 1–2 years of age with a lifespan of 3–7 years, while *Rana nigromaculata* attains sexual maturity at 2 years and has a lifespan of 4–5 years (Lu et al. 2006; Ma et al. 2009; Zhang et al. 2013). The present study findings reveal one distinct pattern of balanced strategy in time allocation among the studied species: most species, including *D. melanostictus* , *O. graminea* , *P. megacephalus* , and *F. multistriata* , allocate approximately 50% of their lifespan to growth and 50% to reproduction. *A. torrentis* and *K. odontotarsus* invest 60% of their lifespan in growth and delay reproduction, and *O. hainanensis* allocates 56% of its lifespan to reproduction, maximizing reproductive output in resource‐rich environments. These strategies enable these species to maintain reproductive success while ensuring adequate survival investment, particularly in environments with consistent resource availability (Stearns 1998; Ellis et al. 2009). This strategy is consistent with the predictions of the “slow‐life” paradigm, wherein species invest in prolonged reproduction to optimize fitness over extended lifespans.

The distinct life history strategies observed in Diaoluoshan reflect adaptations to the ecological pressures of tropical rainforests, including stable climates, abundant resources, and low seasonality. Compared to temperate and subtropical amphibians, which often exhibited shorter lifespans and earlier reproduction due to seasonal variability (Altunışık et al. 2021; Jiang et al. 2022), the tropical anurans in our study demonstrated greater lifespan and variability in maturity timing. This supports the hypothesis that stable environments favor delayed maturity and prolonged reproductive efforts as evolutionary strategies (Stearns 1998; Jonsson et al. 2013; Jonsson and Jonsson 2014).

### 4.3 Conservation Implications and Future Directions

This study uncovers the ecological and evolutionary dynamics of tropical anurans, furnishing critical data for biodiversity conservation and ecosystem management, with a focus on the unique slow‐life strategy of Diaoluoshan anurans to develop evidence‐based conservation strategies. First, their delayed sexual maturity (3–4 years) and extended lifespan (5–9 years) guide the design of habitat protection thresholds, advocating permanent core zones and breeding buffer zones. Second, species‐specific life history trait variations enable targeted monitoring and threat assessment, replacing one‐size‐fits‐all interventions. Third, confirming their slow‐life strategy (in contrast to temperate/subtropical congeners) informs climate change adaptation planning, supporting proactive measures like wildlife corridors via species distribution models. Fourth, preliminary data on understudied species (e.g., *O. hainanensis* , *F. multistriata* ) identify research gaps for priority follow‐up studies. Future research will integrate ecological, genetic and environmental analyses, conduct longitudinal population studies across broader tropical regions, and combine findings with current data to refine conservation strategies, safeguarding the long‐term survival of tropical anuran communities amid global environmental changes.

## 5 Conclusion

Our study provided the first characterization of lifespan and sexual maturity timing in anuran amphibians inhabiting a tropical island rainforest. The observed “slow‐life” strategies—marked by delayed maturation, extended longevity, and a balanced allocation of effort between growth and reproduction—reflected a key adaptation to the stable, resource‐abundant environment of Hainan's tropical forests. These results broadened the framework for understanding life‐history evolution across climatic gradients. Moving forward, integrating demographic modeling with direct evaluations of climate change effects, habitat fragmentation, and disease dynamics will be crucial. Such integrated approaches are needed to translate life‐history insights into proactive conservation measures that safeguard the resilience of tropical amphibian assemblages and the complex ecosystems they support.

## Author Contributions

**Rui Guo:** funding acquisition (equal), investigation (equal), methodology (equal), writing – original draft (equal), writing – review and editing (equal). **Li Zhu:** investigation (equal), methodology (equal). **Longhui Zhao:** data curation (equal), funding acquisition (equal), methodology (equal). **Xiaofei Zhai:** investigation (equal). **Tongliang Wang:** investigation (equal). **Jichao Wang:** project administration (supporting).

## Funding

This work was supported by the National Natural Science Foundation of China (32460252) and the Natural Science Foundation of Hainan Province (323QN232 and 320QN255).

## Ethics Statement

All experiments were carried out according to “Guidance of the Care and Use of Laboratory Animals” approved by the Animal Research Ethics Committee of Hainan Provincial Education Center for Ecology and Environment, Hainan Normal University, China (2019‐007). All field investigations were conducted at the Diaoluo Mountain Diversity of Vertebrate Hainan Observation and Research Station of Hainan Normal University (located in Diaoluo Mountain National Park), with all required field permits obtained in accordance with national and local regulations on wildlife and field research.

## Conflicts of Interest

The authors declare no conflicts of interest.

## Supporting information


**Data S1:** Supporting Information.

**Table S1:** ece373924‐sup‐0002‐Supplementarytable1.docx.

## Data Availability

The data that supports the findings of this study are available in the Supporting Information of this article.
