# Supplementary material for: Study on Life History Strategies of Anurans in Tropical Rainforest: Lifespan and Age at Sexual Maturity
Source: Ecol Evol. 2026 Jun 30;16(7):e73924. doi: 10.1002/ece3.73924 (PMC13319404; doi:10.1002/ece3.73924)
Supplement: Supplementary file 2 — Table S1: ece373924‐sup‐0002‐Supplementarytable1.docx. [file ECE3-16-e73924-s002.docx]

| Species | Longitude E | Latitude N | Elevation (m) | Sex | Sample | Age of sexual maturity (year) | Maximum longevity (year) | References |
| --- | --- | --- | --- | --- | --- | --- | --- | --- |
| *Amolops mantzorum* | 102.93 | 30.57 | 1390 | ♀ | 42 | 2 | 7 | (Liao and Lu,2010) |
| *Amolops mantzorum* | 102.93 | 30.57 | 1390 | ♂ | 24 | 2 | 6 | (Liao and Lu,2010) |
| *Amolops mantzorum* | 102.93 | 30.55 | 1700 | ♀ | 67 | 3 | 9 | (Liao and Lu, 2010) |
| *Amolops mantzorum* | 102.93 | 30.55 | 1700 | ♂ | 76 | 2 | 7 | (Liao and Lu, 2010) |
| *Amolops mantzorum* | 102.92 | 30.53 | 1800 | ♀ | 52 | 3 | 8 | (Liao and Lu,2010) |
| *Amolops mantzorum* | 102.92 | 30.53 | 1800 | ♂ | 21 | 2 | 7 | (Liao and Lu,2010) |
| *Amolops torrentis* | 109.91 | 18.66 | 500-800 | ♂ | 48 | 3 | 5 | Unpublic |
| *Bufo andrewsi* | 102.90 | 30.30 | 760 | ♀ | 32 | 2 | 5 | (Liao et al., 2015) |
| *Bufo andrewsi* | 102.90 | 30.30 | 760 | ♂ | 47 | 1 | 4 | (Liao et al., 2015) |
| *Bufo andrewsi* | 102.64 | 29.35 | 864 | ♂ | 26 | 1 | 5 | (Zhao, 2020) |
| *Bufo andrewsi* | 102.64 | 29.35 | 864 | ♂ | 26 | 1 | 3 | (Jiang et al., 2022) |
| *Bufo andrewsi* | 102.83 | 30.35 | 1000 | ♀ | 26 | 2 | 6 | (Liao et al., 2015) |
| *Bufo andrewsi* | 102.83 | 30.35 | 1000 | ♂ | 44 | 1 | 4 | (Liao et al., 2015) |
| *Bufo andrewsi* | 106.52 | 32.63 | 1351 | ♂ | 25 | 2 | 6 | (Zhao, 2020) |
| *Bufo andrewsi* | 102.92 | 30.53 | 1390 | ♂ | 15 | 2 | 4 | (Liao et al., 2015) |
| *Bufo andrewsi* | 108.52 | 33.56 | 1393 | ♂ | 28 | 2 | 6 | (Jiang et al., 2022) |
| *Bufo andrewsi* | 102.21 | 29.90 | 1477 | ♂ | 21 | 1 | 5 | (Zhao, 2020) |
| *Bufo andrewsi* | 102.21 | 29.90 | 1477 | ♂ | 21 | 1 | 5 | (Jiang et al., 2022) |
| *Bufo andrewsi* | 102.15 | 27.95 | 1522 | ♂ | 23 | 1 | 3 | (Zhao, 2020) |
| *Bufo andrewsi* | 102.15 | 27.95 | 1522 | ♂ | 23 | 1 | 3 | (Jiang et al., 2022) |
| *Bufo andrewsi* | 103.31 | 31.08 | 1524 | ♂ | 30 | 1 | 3 | (Zhao, 2020) |
| *Bufo andrewsi* | 103.31 | 31.08 | 1524 | ♂ | 30 | 1 | 3 | (Jiang et al., 2022) |
| *Bufo andrewsi* | 103.84 | 31.67 | 1553 | ♂ | 19 | 2 | 4 | (Zhao, 2020) |
| *Bufo andrewsi* | 103.84 | 31.67 | 1553 | ♂ | 19 | 2 | 4 | (Jiang et al., 2022) |
| *Bufo andrewsi* | 102.19 | 28.29 | 1632 | ♂ | 28 | 1 | 3 | (Zhao, 2020) |
| *Bufo andrewsi* | 106.55 | 32.66 | 1651 | ♂ | 26 | 1 | 5 | (Zhao, 2020) |
| *Bufo andrewsi* | 106.55 | 32.66 | 1651 | ♂ | 26 | 1 | 5 | (Jiang et al., 2022) |
| *Bufo andrewsi* | 102.93 | 30.55 | 1690 | ♀ | 120 | 3 | 7 | (Liao et al., 2015) |
| *Bufo andrewsi* | 102.93 | 30.55 | 1690 | ♂ | 229 | 2 | 6 | (Liao et al., 2015) |
| *Bufo andrewsi* | 102.47 | 26.74 | 1916 | ♂ | 22 | 2 | 4 | (Zhao, 2020) |
| *Bufo andrewsi* | 102.47 | 26.74 | 1916 | ♂ | 22 | 2 | 4 | (Jiang et al., 2022) |
| *Bufo andrewsi* | 103.17 | 31.03 | 1950 | ♀ | 22 | 3 | 7 | (Zhao, 2020) |
| *Bufo andrewsi* | 103.18 | 31.03 | 1961 | ♂ | 25 | 1 | 4 | (Zhao, 2020) |
| *Bufo andrewsi* | 103.18 | 31.03 | 1961 | ♂ | 25 | 1 | 4 | (Jiang et al., 2022) |
| *Bufo andrewsi* | 102.88 | 25.29 | 1995 | ♂ | 28 | 1 | 4 | (Zhao, 2020) |
| *Bufo andrewsi* | 99.47 | 27.57 | 2028 | ♀ | 27 | 3 | 10 | (Liao et al., 2015) |
| *Bufo andrewsi* | 99.47 | 27.57 | 2028 | ♂ | 167 | 1 | 9 | (Liao et al., 2015) |
| *Bufo andrewsi* | 102.85 | 28.01 | 2050 | ♂ | 30 | 1 | 4 | (Zhao, 2020) |
| *Bufo andrewsi* | 102.04 | 31.30 | 2078 | ♂ | 26 | 2 | 4 | (Zhao, 2020) |
| *Bufo andrewsi* | 102.04 | 31.30 | 2087 | ♂ | 26 | 2 | 4 | (Jiang et al., 2022) |
| *Bufo andrewsi* | 102.95 | 30.53 | 2100 | ♀ | 22 | 3 | 8 | (Liao et al., 2015) |
| *Bufo andrewsi* | 102.95 | 30.53 | 2100 | ♂ | 22 | 3 | 6 | (Liao et al., 2015) |
| *Bufo andrewsi* | 103.32 | 31.32 | 2120 | ♂ | 25 | 2 | 9 | (Liao et al., 2015) |
| *Bufo andrewsi* | 103.21 | 31.42 | 2180 | ♂ | 21 | 1 | 3 | (Zhao, 2020) |
| *Bufo andrewsi* | 99.30 | 27.55 | 2328 | ♂ | 110 | 2 | 9 | (Liao et al., 2015) |
| *Bufo andrewsi* | 100.28 | 26.83 | 2367 | ♂ | 26 | 1 | 4 | (Zhao, 2020) |
| *Bufo andrewsi* | 100.28 | 26.83 | 2367 | ♂ | 26 | 1 | 4 | (Jiang et al., 2022) |
| *Bufo andrewsi* | 102.72 | 30.68 | 2387 | ♂ | 15 | 3 | 9 | (Liao et al., 2015) |
| *Bufo andrewsi* | 99.32 | 27.55 | 2422 | ♀ | 17 | 3 | 7 | (Liao et al., 2015) |
| *Bufo andrewsi* | 99.32 | 27.55 | 2422 | ♂ | 65 | 2 | 7 | (Liao et al., 2015) |
| *Bufo andrewsi* | 104.15 | 32.90 | 2452 | ♀ | 18 | 6 | 11 | (Liao et al., 2015) |
| *Bufo andrewsi* | 104.15 | 32.90 | 2452 | ♂ | 28 | 3 | 8 | (Liao et al., 2015) |
| *Bufo andrewsi* | 99.22 | 27.33 | 2520 | ♂ | 75 | 1 | 8 | (Liao et al., 2015) |
| *Bufo andrewsi* | 102.20 | 28.92 | 2554 | ♀ | 43 | 3 | 6 | (Liao et al., 2015) |
| *Bufo andrewsi* | 102.20 | 28.92 | 2554 | ♂ | 63 | 2 | 5 | (Liao et al., 2015) |
| *Bufo andrewsi* | 104.10 | 32.97 | 2640 | ♀ | 27 | 4 | 11 | (Liao et al., 2015) |
| *Bufo andrewsi* | 104.10 | 32.97 | 2640 | ♂ | 47 | 3 | 11 | (Liao et al., 2015) |
| *Bufo andrewsi* | 99.37 | 27.65 | 2768 | ♂ | 197 | 1 | 11 | (Liao et al., 2015) |
| *Bufo andrewsi* | 101.50 | 29.01 | 2902 | ♂ | 16 | 2 | 4 | (Zhao, 2020) |
| *Bufo andrewsi* | 101.50 | 29.01 | 2902 | ♂ | 16 | 2 | 4 | (Jiang et al., 2022) |
| *Bufo gargarizans* | 104.75 | 30.58 | 300 | ♂ | 56 | 1 | 4 | (Yu and Guo, 2015) |
| *Bufo raddei* | 104.40 | 36.44 | 1428 | ♀ | 96 | 2 | 4 | (Zhang et al., 2018) |
| *Bufo raddei* | 104.40 | 36.44 | 1428 | ♂ | 101 | 2 | 4 | (Guo et al., 2018) |
| *Bufo raddei* | 103.32 | 35.94 | 1624 | ♀ | 141 | 2 | 4 | (Zhang et al., 2018) |
| *Bufo raddei* | 103.32 | 35.94 | 1624 | ♂ | 110 | 2 | 4 | (Guo et al., 2018) |
| *Bufo tibetanus* | 95.68 | 29.88 | 2650 | ♀ | 19 | 4 | 9 | (Xu et al., 2024) |
| *Bufo tibetanus* | 95.68 | 29.88 | 2650 | ♂ | 83 | 3 | 8 | (Xu et al., 2024) |
| *Bufo tibetanus* | 96.75 | 29.50 | 3930 | ♀ | 76 | 5 | 11 | (Xu et al., 2024) |
| *Bufo tibetanus* | 94.36 | 29.65 | 2400-4300 | ♀ | 29 | 4 | 9 | (Li, 2022) |
| *Bufo tibetanus* | 94.36 | 29.65 | 2400-4300 | ♂ | 34 | 4 | 8 | (Li, 2022) |
| *Bufo tibetanus* | 96.75 | 29.50 | 3930 | ♂ | 101 | 4 | 10 | (Xu et al., 2024) |
| *Duttaphrynus himalayanus* | 97.01 | 28.48 | 1770-1805 | ♀ | 56 | 3 | 8 | (Sheng, 2021) |
| *Duttaphrynus himalayanus* | 97.01 | 28.48 | 1770-1805 | ♂ | 80 | 2 | 7 | (Sheng, 2021) |
| *Duttaphrynus melanostictus* | 109.91 | 18.66 | 500-800 | ♀ | 122 | 3 | 6 | Unpublic |
| *Duttaphrynus melanostictus* | 109.91 | 18.66 | 500-800 | ♂ | 80 | 3 | 6 | Unpublic |
| *Duttaphrynus melanostictus* | 97.02 | 28.50 | 1527 | ♀ | 38 | 2 | 7 | (Yuan, 2022) |
| *Duttaphrynus melanostictus* | 97.02 | 28.50 | 1527 | ♂ | 57 | 2 | 6 | (Yuan, 2022) |
| *Feirana quadranus* | 107.70 | 34.06 | 1206 | ♀ | 26 | 5 | 12 | (Xian, 2017) |
| *Feirana quadranus* | 107.70 | 34.06 | 1206 | ♂ | 18 | 5 | 9 | (Xian, 2017) |
| *Feirana taihangnica* | 112.12 | 35.25 | 760-840 | ♀ | 78 | 4 | 12 | (Lu, 2011) |
| *Feirana taihangnica* | 112.12 | 35.25 | 760-840 | ♂ | 43 | 4 | 12 | (Lu, 2011) |
| *Fejervarya multistriata* | 121.45 | 31.19 | 2.19 | ♀ | 160 |  | 3 | (Zhong, 2022) |
| *Fejervarya multistriata* | 121.45 | 31.19 | 2.19 | ♂ | 121 |  | 3 | (Zhong, 2022) |
| *Fejervarya multistriata* | 109.91 | 18.66 | 500-800 | ♂ | 18 | 3 | 6 | unpublic |
| *Hyla annectans chuanxiensis* | 102.93 | 30.55 | 1700 | ♀ | 17 | 2 | 5 | (Liao and Lu, 2010) |
| *Hyla annectans chuanxiensis* | 102.93 | 30.55 | 1700 | ♂ | 31 | 2 | 4 | (Liao and Lu, 2010) |
| *Hyla annectanschuanxiensis* | 102.90 | 30.32 | 800 | ♀ | 16 | 2 | 4 | (Liao and Lu, 2010) |
| *Hyla annectanschuanxiensis* | 102.90 | 30.32 | 800 | ♂ | 33 | 1 | 3 | (Liao and Lu, 2010) |
| *Hylarana guentheri* | 105.37 | 30.38 | 300 | ♀ | 61 | 2 | 6 | (Li et al., 2010) |
| *Hylarana guentheri* | 105.37 | 30.38 | 300 | ♂ | 78 | 1 | 4 | (Li et al., 2010) |
| *Kurixalus odontotarsus* | 109.91 | 18.66 | 500-800 | ♀ | 21 | 3 | 5 | Unpublic |
| *Kurixalus odontotarsus* | 109.91 | 18.66 | 500-800 | ♂ | 66 | 3 | 5 | Unpublic |
| *Nanorana chayuensis* | 97.02 | 28.50 | 1000-1540 | ♀ | 49 | 4 | 7 | (Li, 2022) |
| *Nanorana chayuensis* | 97.02 | 28.50 | 1000-1540 | ♂ | 37 | 4 | 6 | (Li, 2022) |
| *Nanorana parkeri* | 94.48 | 29.57 | 2850-5000 | ♀ | 18 | 4 | 7 | (Li, 2022) |
| *Nanorana parkeri* | 94.48 | 29.57 | 2850-5000 | ♂ | 30 | 3 | 7 | (Li, 2022) |
| *Odorrana graminea* | 109.91 | 18.66 | 500-800 | ♂ | 57 | 3 | 6 | Unpublic |
| *Odorrana hainanensis* | 109.91 | 18.66 | 500-800 | ♀ | 25 | 4 | 9 | Unpublic |
| *Pelophylax plancyi* | 121.45 | 31.19 | 2.19 | ♀ | 95 |  | 5 | (Zhong, 2022) |
| *Pelophylax plancyi* | 121.45 | 31.19 | 2.19 | ♂ | 63 |  | 3 | (Zhong, 2022) |
| *Pelophylax pleuraden* | 102.61 | 27.18 | 1413 | ♀ | 26 | 1.5 | 4.5 | (Lou et al., 2012) |
| *Pelophylax pleuraden* | 102.61 | 27.18 | 1413 | ♂ | 34 | 1.5 | 3.5 | (Lou et al., 2012) |
| *Pelophylax pleuraden* | 102.61 | 27.17 | 1585 | ♀ | 18 | 1.5 | 3.5 | (Lou et al., 2012) |
| *Pelophylax pleuraden* | 102.61 | 27.17 | 1585 | ♂ | 24 | 2.5 | 3.5 | (Lou et al., 2012) |
| *Pelophylax pleuraden* | 102.61 | 27.16 | 1719 | ♀ | 15 | 1.5 | 4.5 | (Lou et al., 2012) |
| *Pelophylax pleuraden* | 102.61 | 27.16 | 1719 | ♂ | 21 | 1.5 | 4.5 | (Lou et al., 2012) |
| *Pelophylax pleuraden* | 102.59 | 27.16 | 1785 | ♂ | 43 | 1.5 | 2.5 | (Lou et al., 2012) |
| *Pelophylax pleuraden* | 102.80 | 24.89 | 1934 | ♀ | 22 | 2 | 4 | (Luo, 2009) |
| *Pelophylax pleuraden* | 102.80 | 24.89 | 1934 | ♂ | 42 | 1 | 4 | (Luo, 2009) |
| *Pelophylax pleuraden* | 102.58 | 27.17 | 1935 | ♀ | 17 | 1.5 | 4.5 | (Lou et al., 2012) |
| *Pelophylax pleuraden* | 102.58 | 27.17 | 1935 | ♂ | 22 | 2.5 | 3.5 | (Lou et al., 2012) |
| *Pelophylax nigromaculatus* | 113.88 | 35.30 | 70-74 | ♀ | 23 | 3 | 8 | (Yang, 2023) |
| *Pelophylax nigromaculatus* | 113.88 | 35.30 | 70-74 | ♂ | 28 | 2 | 6 | (Yang, 2023) |
| *Polypedates megacephalus* | 106.96 | 22.47 | 300-600 | ♂ | 41 | 2 | 5 | (Li, 2011) |
| *Polypedates megacephalus* | 108.74 | 27.77 | 449 | ♂ | 20 | 2 | 5 | (Jin et al., 2017) |
| *Polypedates megacephalus* | 109.91 | 18.66 | 500-800 | ♂ | 77 | 3 | 6 | unpublic |
| *Polypedates megacephalus* | 108.73 | 26.39 | 680 | ♂ | 27 | 2 | 5 | (Jin et al., 2017) |
| *Polypedates megacephalus* | 106.38 | 29.84 | 688 | ♂ | 48 | 2 | 5 | (Jin et al., 2017) |
| *Polypedates megacephalus* | 108.17 | 26.38 | 1300 | ♂ | 41 | 2 | 5 | (Jin et al., 2017) |
| *Polypedates megacephalus* | 110.19 | 24.14 | 1979 | ♂ | 28 | 2 | 5 | (Li, 2011) |
| *Polypedates megacephalus* | 108.78 | 27.84 | 2572 | ♂ | 28 | 1 | 4 | (Li, 2011) |
| *Rana amurensis* | 122.77 | 47.97 | 300 | ♀ | 46 | 1 | 4 | (Liao, 2011) |
| *Rana amurensis* | 122.77 | 47.97 | 300 | ♂ | 45 | 1 | 4 | (Liao, 2011) |
| *Rana amurensis* | 120.82 | 46.72 | 500 | ♀ | 40 | 3 | 7 | (Chen and Lu, 2011) |
| *Rana amurensis* | 120.82 | 46.72 | 500 | ♂ | 36 | 2 | 5 | (Chen and Lu, 2011) |
| *Rana amurensis* | 119.80 | 47.28 | 900 | ♀ | 39 | 3 | 7 | (Chen and Lu, 2011) |
| *Rana amurensis* | 119.80 | 47.28 | 900 | ♂ | 40 | 2 | 6 | (Chen and Lu, 2011) |
| *Rana chensinensis* | 124.02 | 41.07 | 367 | ♀ | 32 | 1 | 4 | （Chen et al., 2011) |
| *Rana chensinensis* | 124.02 | 41.07 | 367 | ♂ | 28 | 1 | 5 | （Chen et al., 2011) |
| *Rana chensinensis* | 112.77 | 36.57 | 567 | ♀ | 33 | 1 | 4 | (Ma et al., 2009) |
| *Rana chensinensis* | 112.77 | 36.57 | 567 | ♂ | 53 | 1 | 4 | (Ma et al., 2009) |
| *Rana chensinensis* | 112.33 | 39.67 | 1400 | ♀ | 21 | 1 | 3 | （Lu et al., 2006) |
| *Rana chensinensis* | 112.33 | 39.67 | 1400 | ♂ | 27 | 1 | 2 | （Lu et al., 2006) |
| *Rana chensinensis* | 112.90 | 36.58 | 1470 | ♂ | 38 | 1 | 4 | (Ma et al., 2009) |
| *Rana chensinensis* | 112.45 | 37.80 | 1700 | ♀ | 21 | 1 | 6 | (Ma et al., 2009) |
| *Rana chensinensis* | 112.33 | 39.67 | 1700 | ♀ | 20 | 1 | 6 | （Lu et al., 2006) |
| *Rana chensinensis* | 112.45 | 37.80 | 1700 | ♂ | 25 | 1 | 5 | (Ma et al., 2009) |
| *Rana chensinensis* | 112.33 | 39.67 | 1700 | ♂ | 22 | 2 | 3 | （Lu et al., 2006) |
| *Rana chensinensis* | 112.33 | 39.67 | 2000 | ♀ | 20 | 2 | 7 | （Lu et al., 2006) |
| *Rana chensinensis* | 112.33 | 39.67 | 2000 | ♂ | 24 | 1 | 3 | （Lu et al., 2006) |
| *Rana chensinensis* | 106.23 | 35.68 | 2122 | ♂ | 25 | 2 | 5 | (Xian, 2017) |
| *Rana kukunoris* | 101.63 | 36.65 | 2297 | ♂ | 17 | 2 | 4 | (Yu et al., 2021) |
| *Rana kukunoris* | 101.33 | 36.67 | 2594 | ♂ | 39 | 2 | 4 | (Yu et al., 2021) |
| *Rana kukunoris* | 103.18 | 34.63 | 2706 | ♂ | 50 | 2 | 4 | (Yu et al., 2021) |
| *Rana kukunoris* | 101.43 | 36.48 | 2789 | ♀ | 43 | 3 | 6 | (Yu et al., 2021) |
| *Rana kukunoris* | 101.43 | 36.48 | 2789 | ♂ | 53 | 2 | 5 | (Yu et al., 2021) |
| *Rana kukunoris* | 103.93 | 33.05 | 2909 | ♀ | 68 | 2 | 8 | (Zhang et al., 2023) |
| *Rana kukunoris* | 103.93 | 33.05 | 2909 | ♂ | 25 | 2 | 3 | (Zhang et al., 2023) |
| *Rana kukunoris* | 103.00 | 36.90 | 2999 | ♀ | 25 | 3 | 7 | (Yu et al., 2021) |
| *Rana kukunoris* | 103.00 | 36.90 | 2999 | ♂ | 84 | 2 | 6 | (Yu et al., 2021) |
| *Rana kukunoris* | 103.23 | 34.78 | 3036 | ♀ | 51 | 3 | 7 | (Yu et al., 2021) |
| *Rana kukunoris* | 103.23 | 34.78 | 3036 | ♂ | 71 | 3 | 5 | (Yu et al., 2021) |
| *Rana kukunoris* | 102.68 | 34.48 | 3049 | ♀ | 18 | 3 | 6 | (Yu et al., 2021) |
| *Rana kukunoris* | 102.68 | 34.48 | 3049 | ♂ | 19 | 3 | 5 | (Yu et al., 2021) |
| *Rana kukunoris* | 102.45 | 34.77 | 3100 | ♀ | 51 | 3 | 8 | （Chen et al., 2011) |
| *Rana kukunoris* | 102.45 | 34.77 | 3100 | ♂ | 121 | 3 | 6 | （Chen et al., 2011) |
| *Rana kukunoris* | 102.68 | 34.37 | 3233 | ♀ | 30 | 3 | 5 | (Yu et al., 2021) |
| *Rana kukunoris* | 102.68 | 34.37 | 3233 | ♂ | 24 | 2 | 4 | (Yu et al., 2021) |
| *Rana kukunoris* | 102.45 | 34.77 | 3400 | ♀ | 42 | 4 | 8 | （Chen et al., 2011) |
| *Rana kukunoris* | 102.45 | 34.77 | 3400 | ♂ | 63 | 3 | 7 | （Chen et al., 2011) |
| *Rana kukunoris* | 102.32 | 34.28 | 3441 | ♀ | 42 | 3 | 8 | (Yu et al., 2021) |
| *Rana kukunoris* | 102.32 | 34.28 | 3441 | ♂ | 73 | 3 | 7 | (Yu et al., 2021) |
| *Rana kukunoris* | 102.95 | 33.58 | 3450 | ♀ | 25 | 1 | 5 | (Li, 2011) |
| *Rana kukunoris* | 102.95 | 33.58 | 3450 | ♂ | 33 | 1 | 7 | (Li, 2011) |
| *Rana limnocharis* | 105.37 | 30.38 | 310 | ♀ | 42 | 1 | 4 | （Liao et al., 2011) |
| *Rana limnocharis* | 105.37 | 30.38 | 310 | ♂ | 150 | 1 | 3 | （Liao et al., 2011) |
| *Rana limnocharis* | 102.90 | 30.32 | 800 | ♀ | 20 | 1 | 4 | （Liao et al., 2011) |
| *Rana limnocharis* | 102.90 | 30.32 | 800 | ♂ | 22 | 1 | 3 | （Liao et al., 2011) |
| *Rana nigromaculata* | 105.37 | 30.38 | 300 | ♀ | 32 | 2 | 5 | (Liao et al., 2010) |
| *Rana nigromaculata* | 105.37 | 30.38 | 300 | ♂ | 32 | 2 | 4 | (Liao et al., 2010) |
| *Rana nigromaculata* | 112.77 | 36.57 | 800 | ♀ | 24 | 2 | 4 | (Liao et al., 2010) |
| *Rana nigromaculata* | 112.77 | 36.57 | 800 | ♂ | 22 | 2 | 4 | (Liao et al., 2010) |
| *Rana omeimontis* | 104.55 | 28.78 | 308 | ♂ | 63 | 1 | 4 | (Liu et al., 2012) |
| *Rana sauteri* | 120.61 | 23.43 | 330 | ♀ | 35 | 1 | 3 | （HSU et al., 2014) |
| *Rana sauteri* | 120.61 | 23.43 | 330 | ♂ | 120 | 1 | 3 | （HSU et al., 2014) |
| *Rana sauteri* | 120.65 | 23.45 | 630 | ♀ | 41 | 1 | 4 | （HSU et al., 2014) |
| *Rana sauteri* | 120.65 | 23.45 | 630 | ♂ | 732 | 1 | 4 | （HSU et al., 2014) |
| *Rana sauteri* | 120.69 | 23.48 | 970 | ♀ | 24 | 1 | 4 | （HSU et al., 2014) |
| *Rana sauteri* | 120.69 | 23.48 | 970 | ♂ | 169 | 1 | 4 | （HSU et al., 2014) |
| *Rana sauteri* | 120.72 | 23.48 | 1380 | ♀ | 25 | 1 | 5 | （HSU et al., 2014) |
| *Rana sauteri* | 120.72 | 23.48 | 1380 | ♂ | 242 | 1 | 5 | （HSU et al., 2014) |
| *Rana sauteri* | 120.81 | 23.52 | 2100 | ♂ | 54 | 2 | 5 | （HSU et al., 2014) |
| *Rana sauteri* | 120.82 | 23.51 | 2320 | ♂ | 88 | 2 | 5 | （HSU et al., 2014) |
| *Rana swinhoana* | 121.67 | 24.85 | 200 | ♀ | 28 | 2 | 7 | (Lai et al., 2005) |
| *Rana swinhoana* | 121.67 | 24.85 | 200 | ♂ | 15 | 2 | 6 | (Lai et al., 2005) |
| *Rana swinhoana* | 121.18 | 24.13 | 1600 | ♀ | 22 | 4 | 11 | (Lai et al., 2005) |
| *Rana swinhoana* | 121.18 | 24.13 | 1600 | ♂ | 43 | 2 | 7 | (Lai et al., 2005) |
| *Rhacophorus omeimontis* | 102.90 | 30.30 | 760 | ♀ | 15 | 1 | 4 | (Liao and Lu,2011) |
| *Rhacophorus omeimontis* | 102.90 | 30.30 | 760 | ♂ | 38 | 2 | 5 | (Liao and Lu,2011) |
| *Rhacophorus omeimontis* | 102.93 | 30.55 | 1690 | ♀ | 27 | 3 | 8 | (Liao and Lu,2011) |
| *Rhacophorus omeimontis* | 102.93 | 30.55 | 1690 | ♂ | 141 | 2 | 6 | (Liao and Lu,2011) |
| *Rhacophorus omeimontis* | 102.83 | 30.35 | 1000 | ♂ | 23 | 2 | 5 | (Liao and Lu,2011) |
| *Scutiger boulengeri* | 92.45 | 20.91 | 4351 | ♀ | 26 | 6 | 15 | (An, 2019) |
| *Scutiger boulengeri* | 92.45 | 20.91 | 4351 | ♂ | 63 | 5 | 13 | (An, 2019) |
